# The Effect of Physical Activity on Sleep Quality and Sleep Disorder: A Systematic Review

**DOI:** 10.7759/cureus.43595

**Published:** 2023-08-16

**Authors:** Majd A Alnawwar, Meiral I Alraddadi, Rafaa A Algethmi, Gufran A Salem, Mohammed A Salem, Abeer A Alharbi

**Affiliations:** 1 Pulmonary Medicine, King Fahad Hospital, Madinah, SAU; 2 Medicine, King AbdulAziz University, Jeddah, SAU; 3 Medicine, Ibn Sina National College for Medical Studies, Jeddah, SAU; 4 Medicine, King Salman Bin Abdulaziz Medical City, Madinah, SAU; 5 Pulmonary Medicine/Sleep Medicine, King Fahad Hospital, Madinah, SAU

**Keywords:** insomnia, sleep disorder, sleep quality, health benefit, physical exercise, physical activity

## Abstract

Regular physical activity has several health benefits, including improved sleep quality and symptoms of sleep disorders. With the known benefits of moderate-intensity activities to sleep quality and a growing interest in using physical activity as a treatment approach for different sleep disorders, we conducted a systematic review to provide evidence-based data on the association between physical activity and sleep. A systematic search was carried out in PubMed, Embase, MEDLINE (Medical Literature Analysis and Retrieval System Online), Google Scholar, and Scopus, using predetermined search terms (Medical Subject Headings (MeSH) terms) and keywords. The included studies focused on exploring the effect of physical activity on sleep quality and sleep disorders or the association between physical activity and sleep outcomes. Relevant data were extracted, and the quality of the studies was evaluated using suitable methods. The collected findings were synthesized and discussed. The findings of this systematic review have potential implications for healthcare, public health policies, and health promotion.

## Introduction and background

Sleep promotes memory consolidation and learning while allowing the body to recover and restore itself [1]. However, many people struggle with sleep issues like insomnia, sleep apnea, and restless legs syndrome. One strategy to enhance sleep quality and lower the risk of sleep disorders is through physical activity, which has a reciprocal relationship with sleep quality [2].

The most significant predictor of sleep status is sleep quality, whose disruption is characterized by difficulty falling or staying asleep and the frequency of nightly awakenings [3,4]. The most prevalent sleep quality problem is insomnia, defined as chronic dissatisfaction with sleep quantity or quality, accompanied by difficulty falling asleep, numerous overnight awakenings with difficulty returning to sleep, and/or waking up earlier than desired [5]. Studies showed that insomnia prevalence rates range from 10% to 30%, and in some cases, even as high as 50-60% [6-8]. This sleep disorder is particularly prevalent among older adults, females, and individuals with medical and mental health conditions. Poor sleep quality and sleep disorders impair attention and memory, negatively affecting physical, psychological, and social interaction [9,10].

Physical activity can help you sleep better in a variety of ways. First, it increases the production of melatonin, a hormone that regulates sleep-wake cycles [11]. As a result, physical activity can assist in falling asleep faster and sleeping better. Second, physical activity reduces stress, which is a typical impediment to falling and staying asleep. Third, physical activity improves mood, leading to increased enthusiasm for physical exercise and a positive feedback loop [12]. It was shown that active people had higher levels of positive affect and tranquility during exercise and lower levels of negative affect and tiredness [12,13]. Finally, physical activity helps to regulate body temperature, which is necessary for falling asleep, as an increase in body temperature during physical activity aids the eventual drop 30-90 minutes post-exercises, facilitating easier sleepiness [14].

There is a growing research interest in the effects of physical activity on sleep. Studies have shown that regular physical activity can improve sleep quality and duration [15-18]. Scientific literature shows that adults who exercised for at least 30 minutes a day slept an average of 15 minutes longer than those who did not exercise [19]. Other studies have shown that physical activity can help to reduce sleep disorders, such as insomnia, daytime sleepiness, and sleep apnea [15,19,20]. Moreover, studies have found that moderate-intensity exercise can improve sleep quality in insomnia patients. It was found that patients with insomnia who exercised for 30 minutes three times per week for eight weeks experienced improved sleep quality [19]. In addition, moderate-intensity aerobic exercise improves sleep among patients with insomnia [21]. We conducted a systematic review to evaluate the effect of physical activity on sleep quality and sleep disorders, such as insomnia. This systematic review provides evidence-based data on the association between physical activity and sleep quality and disorders, which could affect healthcare practices, public health policies, and individual lifestyle choices, promoting sleep health and improving overall well-being for individuals and communities.

## Review

Methods

This is a systematic review following the guidelines provided by the Preferred Reporting Items for Systematic Reviews and Meta-Analyses (PRISMA) [22] to identify pertinent articles published between 2013 and 2023 that address the effect of physical activity on sleep quality and sleep disorder.

Search Strategy

We systematically searched these electronic databases: PubMed, Embase, MEDLINE (Medical Literature Analysis and Retrieval System Online), Google Scholar, and Scopus. The search terms were chosen, combining Medical Subject Headings (MeSH terms) and keywords, including "physical exercise," "physical activity," "sleep quality," "sleep disorder," "insomnia," "sleep apnea," and "restless legs syndrome." We combined these terms using Boolean operators ("AND," "OR") to create relevant search queries. We conducted manual searches of the reference lists of identified articles to find any additional studies that might be relevant. Only studies conducted and published within the past decade were considered to incorporate the latest medical research and technology advancements.

Study Selection

Two independent reviewers screened the identified studies based on their titles and abstracts. Eligibility criteria were: randomized controlled trials (RCTs), observational studies, intervention studies, systematic reviews, and meta-analyses that investigated the relationship between physical exercise and sleep quality or sleep disorder outcomes. We considered all studies involving human participants of any age, gender, or health condition, and we did not restrict countries or study settings. However, all articles published more than 10 years ago and in languages other than English were excluded. We also excluded theses, editorials, letters to the editor, commentary, opinion articles, narrative and scoping reviews, and any other article published in non-peer-review journals.

**Figure 1:**
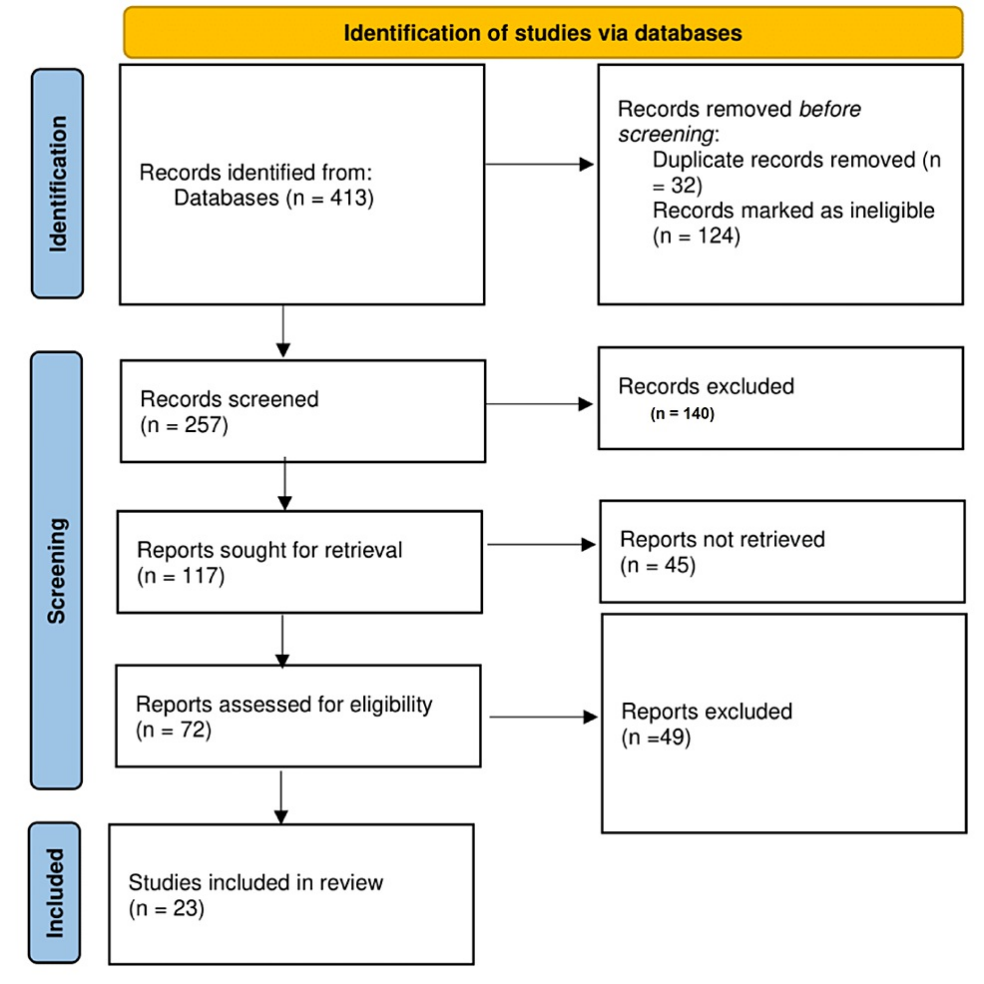
Flow diagram for the selection of studies included in our systematic review

We retrieved and reassessed full-text articles of potentially relevant studies for inclusion criteria. Disagreements between reviewers were resolved through discussion, and a third reviewer intervened if necessary. Figure 1 shows the selection process of the studies included.

Data Extraction and Quality Assessment

Two reviewers independently extracted data using a predefined extraction form, recording these characteristics: authors, publication year, study design, and summary of findings. The quality of included studies was assessed using appropriate tools based on the study design. For cohort studies, we used the Newcastle-Ottawa Scale (NOS) [23], while the Cochrane Collaboration's Risk of Bias tool (CCRB) was used for intervention studies [24]. The NOS, with a scale of 0 to 9, is used to assess the quality of cohort studies. Except for comparability, which allowed for two points, each NOS item could only receive one point, with a minimum score of zero [23]. The CCB contains seven components: random sequence generation, allocation concealment, blinding of participants and people, blinding of result evaluation, completeness of outcome data, report selection, and additional biases [24]. The risk of bias for the study was graded for each item in three categories: low, high, or uncertain risk of bias. The Agency for Healthcare Research and Quality Scale (AHRQ), with scores ranging from 0 to 11, was used to assess the quality of cross-sectional research, with higher ratings showing better research methodology [25]. Two reviewers independently evaluated the risk of bias in the studies. A third reviewer was consulted for assistance when discrepancies could not be resolved.

Data Synthesis

We synthesized the findings by summarizing and categorizing them based on key themes identified in the included studies and presenting them in Table 1. Considering the strength of evidence, consistency of findings, and limitations of the included studies, we discussed the effect of physical activity on sleep quality and sleep disorder. Most sleep outcome measurements reported in studies are composites of sleep quality and quantity rather than measures of a single sleep feature. As a result, the impacts on sleep quantity and quality could not be reported separately.

RESULTS

Database search gave 413 articles initially; articles not written in English, published earlier than 2013, and duplicates were removed and this left us with 257 titles. After removing articles that were theses, editorials, letters to the editor, commentary, opinion articles, narrative and scoping reviews, and any other article published in non-peer-review journals, we were left with 117 articles. Of these, the full text of 45 articles could not be retrieved. The full-text versions of the remaining 72 studies were subjected to thorough scanning. The titles, abstracts, and full texts of only 23 studies were found suitable and they were included in our review (Table 1). The majority of included studies (n=8) were cross-sectional, three were systematic reviews, two were systematic reviews and meta-analyses, three were only meta-analyses, three were longitudinal studies, one was both cross-sectional and longitudinal, one was a randomized clinical trial, one was a cohort, and the last one was a quasi-experimental study.

**Table 1:** Characteristics of the included studies PSQI: Pittsburgh Sleep Quality Index; SMD: Standardized mean difference; CI: Confidence interval; I^2^: Proportion of variation

Authors	Year pf publication	Title	Study design	Summary of findings
Wunsch et al. [3]	2017	The effect of physical activity on sleep quality, well-being, and affect in academic stress periods	Longitudinal study	Physical activity positively impacts sleep quality, wellness, and mood during academic stress periods, influenced by the intensity of activity during the stressful period.
Wendt et al. [26]	2022	Short-term effect of physical activity on sleep health: a population-based study using accelerometry	Prospective cohort	The means of a seep time window, total sleep time, and sleep percent were 443.6 minutes/day, 371.1 minutes/day, and 84%, respectively. The amount of time spent engaging in moderate to intense physical exercise in the morning and the afternoon did not significantly correlate with sleep quality. However, men's morning light physical exercise of 10 minutes per day added 2.56 minutes per day to their overall daily sleep time. Ten minutes per day of gentle exercise in the morning increased women's sleep percentage by 0.15 points. Additionally, women's percentage of sleep was raised by 0.09 points by afternoon light exercise. Notably, both intensity and sex of nighttime physical activity showed a negative relationship with sleep characteristics.
Wang and Boros [13]	2018	The effect of physical activity on sleep quality: a systematic review	Systematic review	Moderate physical activity enhances sleep quality more effectively than vigorous exercise. Additionally, young and old individuals benefit from moderate physical activity regarding sleep quality.
Zhao et al. [27]	2023	Physical activity and sleep quality association in different populations: a meta-analysis	Meta-analysis	Young people's sleep did not appear to be affected by physical exercise; however, children, middle-aged, and elderly individuals were all impacted. High-intensity exercise did not appear to significantly affect the quality of sleep, although moderate exercise did.
Vanderlinden et al. [28]	2020	Effects of physical activity programs on sleep outcomes in older adults: a systematic review	Systematic review	Among older adults, moderate-intensity exercise three times a week for 12 weeks up to six months leads to the most substantial improvements in sleep quality. Single exercise types like Baduanjin, tai chi, and the silver yoga program, whether single or combined, exhibited a higher proportion of significant effects on sleep outcomes compared to other types.
Takács and Török [16]	2019	The relationship between daily physical activity, subjective sleep quality, and mood in sedentary Hungarian adults: a longitudinal within-subjects study	A longitudinal within-subjects study	Daily physical activity and sleep/mood are associated. They found a U-shaped relationship between sleep duration, sleep quality, morning feelings, and steps/day. Increasing steps/day reduces stress and daytime sleepiness while improving sleep efficiency. However, sleep efficiency, daytime sleepiness, and sleep duration do not demonstrate any association.
Štefan et al. [4]	2018	Associations between sleep quality and its domains and insufficient physical activity in a large sample of Croatian young adults: a cross-sectional study	Cross-sectional	Insufficient physical activity is associated with poor sleep quality, more sleep disturbances, more than 60 minutes of sleep latency, less than seven-hour sleep duration, use of sleep medication, and daytime dysfunction
Silva et al. [29]	2022	Effect of physical exercise on sleep quality in elderly adults: a systematic review with a meta-analysis of controlled and randomized studies	A systematic review with a meta-analysis of controlled and randomized studies	Physical exercise significantly enhances sleep quality. Physical activity had a positive impact on both the insomnia group (SMD: -0.57; 95% CI: -0.73 to -0.4; p < 0.001; I^2^ = 53%), non-insomnia group (SMD: -0.61; 95% CI: -0.75 to -0.47; p < 0.00001; I^2^ = 73%), and both groups combined (SMD: -0.59; 95%CI: -0.70 to -0.49; p < 0.0001, I^2^ = 68%).
Santos et al. [30]	2023	Relationship between free‑time physical activity and sleep quality in Brazilian university students	Cross-sectional study	Over half (52.2%) did not engage in free time physical activity, and 75.6% experienced poor sleep quality. In the adjusted analysis, practicing free-time physical activity four to seven times a week was associated with better sleep quality. Students who practiced free-time physical activity scored lower in global PSQI, subjective sleep quality, sleep duration, sleep disturbances, and daytime dysfunction than non-practitioners, indicating a positive impact.
Rosa et al. [18]	2021	Effect of Different Sports Practice on Sleep Quality and Quality of Life in Children and Adolescents: Randomized Clinical Trial	Randomized clinical trial	Physical activity improves the sleep quality and lives of children and adolescents. Physical exercises like Judo (P = 0.032) and ball games (P = 0.005) significantly improved sleep quality in the participants. Additionally, these activities significantly increased perceptions of health and physical activity (judo: mean = 6.9, ball games: mean = 8.91), autonomy (judo: mean = 5.81, ball games: mean = 5.00), friends and social support (judo: mean = 2.83, ball games: mean = 12.00), and reduced provocation and bullying (judo: mean = 10.21, ball games: mean = 2.14).
Murray et al. [31]	2017	The relationship between sleep, time of physical activity, and time outdoors among adult women	Cross-sectional study	A significant interaction (p = 0.04) between moderate-to-vigorous physical activity and total sleep time but not sleep efficiency. Afternoon outdoor time was associated with lower sleep efficiency but did not affect total sleep time.
Mahfouz et al. [32]	2020	Association between sleep quality and physical activity in Saudi Arabian university students	Cross-sectional analytical study	Almost two-thirds of participants reported experiencing poor sleep quality and were physically inactive. 53.4% of students were stressed. Sleep quality significantly correlated with physical activity status (p = 0.009). Sleep duration, daytime dysfunctions, and global PSQI were associated with physical activity levels (p < 0.05 for all). There was a significant association between physical activity and good sleep quality (OR = 1.72, 95%CI: 1.15-2.56, p = 0.008).
Lakshmi Narayana et al. [33]	2023	A cross-sectional study on the effect of physical activity on improving sleep quality among young adults	Cross-sectional study	There was a moderate inverse association between physical activity and PSQI; higher reported physical activity was linked to better sleep quality and low physical activity was associated with poor sleep.
Kredlow et al. [34]	2015	The effects of physical activity on sleep: a meta-analytic review	Meta-analytic review	Consistent physical exercise has slightly positive impacts on total sleep time and efficiency, modest positive effects on sleep onset latency, and notable improvements in sleep quality. Factors like gender, age, baseline physical activity level, exercise type, timing, duration, and adherence influence these effects. However, no significant moderating effects were observed for exercise intensity and aerobic or anaerobic categories.
Kaur et al. [35]	2020	Effect of physical activity on perceived stress, sleep quality, and subjective happiness during middle age	Cross-sectional study	The study results showed a significant impact of physical activity on perceived stress (F = 3.34; p < 0.05) and sleep quality (F = 387.036; p < 0.01). However, physical activity did not significantly affect subjective happiness for both male and female participants.
Inoue et al. [20]	2013	Does habitual physical activity prevent insomnia? a cross-sectional and longitudinal study of elderly Japanese	Cross-sectional and longitudinal study	Regular physical activity was associated with a decreased occurrence of insomnia. Frequent physical activity, particularly in cases of difficulty maintaining sleep, also reduced the likelihood of developing insomnia. For elderly individuals with good mobility and no preexisting health conditions, engaging in physical activity five or more days per week may be beneficial in reducing insomnia.
Holfeld and Ruthig [36]	2014	A longitudinal examination of sleep quality and physical activity in older adults	Longitudinal study	Higher initial sleep quality was linked to increased subsequent physical activity, independent of prior physical activity. Initial physical activity did not predict later sleep quality after considering prior sleep quality.
Ezati et al. [37]	2020	The effect of regular aerobic exercise on sleep quality and fatigue among female student dormitory residents	A quasi-experimental study	There was a significant association between aerobic exercise and sleep quality after 4-week and 8-week interventions (p < 0.001 and p < 0.0001, respectively). Moreover, aerobic exercise significantly decreased the overall fatigue score (p < 0.001).
Dubinina et al. [38]	2021	Physical activity is associated with sleep quality: results of the ESSE-RF epidemiological study	Cross-sectional study	Engaging in excessively frequent and intense physical activities has been linked to difficulties initiating sleep, making them potential risk factors for insomnia. Specifically, having a high physical load six or more times a week was identified as an independent risk factor for trouble falling asleep (OR = 1.9; 95%CI= 1.4–3.4, p = 0.001). However, no significant association was found between leisure-time walking and sleep characteristics.
Cheval et al. [39]	2021	The association between physical activity and cognitive function is partly explained by better sleep quality.	Cross-sectional study	The findings revealed a positive correlation between physical activity and improved sleep quality, which, in turn, positively influenced cognitive function.
Alkhaldi et al. [40]	2023	Effect of nighttime exercise on sleep quality among the general population in Riyadh, Saudi Arabia: a cross-sectional study	Cross-sectional study	Engaging in vigorous evening physical exercise sessions lasting more than 90 minutes was significantly and positively correlated with poor sleep quality, as indicated by a high PSQL score (r = 0.25, p = 0.038). Similarly, moderate evening physical exercise sessions lasting over 90 minutes also showed a significant positive correlation with poor sleep quality (r = 0.30, p = 0.025).
Alfaro-Castro et al. [41]	2021	Impact of moderate physical exercise on sleep disorders in patients with fibromyalgia	Systematic review	Moderate physical exercise has a positive impact on reducing sleep disorders in patients with fibromyalgia. There was an effectiveness of high-intensity interval exercises in improving sleep outcomes in these patients.
Xie et al. [15]	2021	Effects of exercise on sleep quality and insomnia in adults: a systematic review and meta-analysis of randomized controlled trials	Systematic review and meta-analysis	Physical and mind-body exercise interventions led to similar improvements in subjective sleep quality. Interestingly, it was observed that short-term interventions (lasting three months or less) showed a significantly greater reduction in sleep disturbances compared to long-term interventions (lasting more than three months).

The association between physical activities and sleep quality was exclusively reported in 18 articles [3,4,13,16,18,26-28,30-33,35-37,39,40], while five articles reported an association between physical activities and insomnia and other sleep disorders [15,20,29,38,41]. In all articles, physical activity was positively associated with sleep quality, which indicates that physical activities improve sleep quality. These articles showed that physical activities reduce the severity of insomnia and other sleep disorders.

One study reported that insufficient physical activity is positively associated with poor sleep quality, sleep disturbances, more than 60 minutes of sleep latency, less than seven-hour sleep duration, use of sleep medication, and daytime dysfunction [4]. Similarly, a study on university students found that sleep duration, daytime dysfunctions, and the global Pittsburgh Sleep Quality Index (PSQI), which measures sleep quality, were associated with physical activity levels (p < 0.05 for all) [32]. This was also confirmed by another study on young adults [33].

Effect of Physical Activity Type and Intensity

One study found that exercises performed by a single person, like Baduanjin, tai chi, and silver yoga had more significant effects on sleep outcomes than other types [28]. The association between physical activity intensity and sleep quality was reported by seven articles [3,13,26-28,31,40]. Wunsch et al. found the association between physical activity and sleep quality to be influenced by the intensity of the activity [3]. It has also been reported that moderate-intensity physical activity is more effective than vigorous exercise in enhancing sleep quality for young and old individuals [13]. This aligns with another study showing that moderate exercise was associated with improved sleep quality, and high-intensity exercise did not significantly affect sleep quality [27]. However, other studies found a significant interaction (p = 0.04) between moderate-to-vigorous physical activity and total sleep time but not sleep efficiency [31]. Regarding high-intensity physical activities, one study even reported that engaging in vigorous evening physical exercise sessions lasting more than 90 minutes was significantly and positively correlated with poor sleep quality, indicated by a high PSQI score (r = 0.25, p = 0.038) [40].

Effect of Physical Activity Duration and Time

The duration of physical exercises was found to influence the effect on sleep quality in two studies [26,40]. In addition to reporting a negative association between more than 90 minutes of high-intense physical activity and sleep quality, it was found that morning light physical exercise of 10 minutes per day added 2.56 minutes per day to their overall daily sleep time for men, while it increased sleep percentage by 0.15 points for women [40]. Additionally, women's percentage of sleep was raised by 0.09 points by afternoon light exercise, and both intensity and gender showed a negative relationship between nighttime physical activity and sleep quality [26].

Effect of Physical Activity Frequency and Consistency

Physical activity frequency impacts sleep quality, as reported by five studies [16,28,30,37,38]. A study on students found that 52.2% did not engage in free time physical activity, and 75.6% experienced poor sleep quality. For these students, practicing free-time physical activity, especially aerobic exercise, four to seven times a week was associated with better sleep quality after four-week and eight-week interventions (p < 0.001 and p < 0.0001, respectively) [37]. One systematic review concluded that moderate-intensity exercise three times a week for older adults for 12 weeks up to six months led to improved sleep quality [28]. Another study reported that daily physical activity and sleep quality were associated [16]. This study found that increasing the steps done per day reduces stress and daytime sleepiness while improving sleep efficiency.

Kredlow et al., in their meta-analysis, reported that consistent physical exercise had slight positive impacts on total sleep time and efficiency, modest positive effects on sleep onset latency, and significant effects on sleep quality improvement [34]. This study showed that factors like gender, age, baseline physical activity level, exercise type, timing, duration, and adherence influence these effects on sleep quality. However, no significant moderating effects were observed for exercise intensity and aerobic or anaerobic categories. This contrasts with a quasi-experimental study that showed a significant association between regular aerobic exercises and improved sleep quality [37].

Effect of Physical Activity on Sleep Disorders

Of the articles reporting associations between physical activities and insomnia and other sleep disorders [15,20,29,38,41], one reported that physical exercise had a positive impact on both the insomnia group of elderly adults (standardized mean difference (SMD): -0.57; 95%CI: -0.73 to -0.4; p < 0.001; I2 = 53%), non-insomnia group (SMD: -0.61; 95% CI: -0.75 to -0.47; p < 0.00001; I2 = 73%), and both groups combined (SMD: -0.59; 95%CI: -0.70 to -0.49; p < 0.0001, I2 = 68%) [29]. A study conducted on adults with insomnia found that both physical exercise and mind-body exercise interventions led to similar improvements in subjective sleep quality, and short-term interventions (lasting three months or less) showed a significantly greater reduction in sleep disturbances compared to long-term interventions (lasting more than three months) [15]. Another study reported that frequent physical activity and engaging in physical activity five or more days per week may be beneficial in reducing insomnia [20]. However, another study found that excessively frequent (six or more times a week) and intense physical activities were associated with difficulty initiating sleep, making them potential risk factors for insomnia (OR = 1.9; 95%CI= 1.4-3.4; p = 0.001) [38].

Regarding other disorders, one article reported that moderate physical exercise has a positive impact on reducing sleep disorders in patients with fibromyalgia and found the effectiveness of high-intensity interval exercises in improving sleep outcomes in these patients [41]. Cheval et al., in their study, found a positive correlation between physical activity and improved sleep quality, which, in turn, positively influenced cognitive function among middle-aged and older populations [39].

Three articles also found physical activity to positively impact sleep quality, wellness, good mood, reduced stress, and fatigue [3,35,37]. Though most studies in our systematic review were conducted on adults, one meta-analysis that included articles about children in addition to adults found that young people’s sleep quality did not get affected by physical exercise while the sleep of children, middle-aged, and elderly individuals was impacted [27].

Lastly, a longitudinal study examining sleep quality and physical activity in older adults found that higher initial sleep quality was also linked to increased subsequent physical activity, independent of prior physical activity. However, this study found that initial physical activity did not predict later sleep quality after considering prior sleep quality [36].

Discussion

Sleep quality plays a vital role in preserving overall health and wellness. Sufficient and high-quality sleep is indispensable for cognitive functioning, emotional balance, and physical well-being [2]. Over time, scientists have investigated several non-pharmacological methods to enhance sleep quality and address sleep disorders, and one among these approaches is engaging in physical activity [2,6,42]. Numerous studies have examined how physical activity affects sleep results, resulting in a growing body of evidence indicating the potential advantages of physical exercise in enhancing sleep quality and possible effects on sleep disorders. Therefore, this systematic review aimed to compile data from relevant studies to synthesize the recent evidence on the effect of physical activity on sleep quality and sleep disorders.

This systematic review found that physical activity was positively associated with sleep quality among different categories of populations. This is consistent with the World Health Organization (WHO) and Centers for Disease Control and Prevention (CDC) recommending engaging in physical activities to improve sleep quality [43,44]. The association between physical activity and sleep quality can be attributed to several mechanisms, including the release of endorphins, which can lower stress and anxiety, resulting in improved relaxation and better sleep, regulation of circadian rhythms, and a rise in body temperature followed by a subsequent decrease helping in initiating sleep [14,45]. Furthermore, exercise stimulates the release of neurotransmitters such as serotonin and norepinephrine, which are involved in mood regulation and relaxation, perhaps assisting in better sleep start and maintenance [46].

This systematic review found that most studies reported a positive impact of moderate intensity of physical activities on sleep quality. However, high-intensity physical activities were mainly found to lead to poor sleep quality, indicating that intensity plays a role in influencing which effect physical activities have on sleep quality. This aligns with studies that show that moderate-intensity physical activities improve sleep quality by decreasing sleep latency, increasing total sleep time [19], and decreasing pre-sleep anxiety [21]. Studies have also found that moderate physical activities enhance overall sleep length, decrease sleep latency, and reduce the number of nightly awakenings [3,4]. Most studies in our review that reported on high-intensity physical activities found that it exerted an opposite effect on sleep quality, leading to difficulties in falling asleep. Similarly, a systematic review and meta-analysis has found that high-intensity physical exercises were more likely to disrupt sleep than moderate-intensity physical exercises, primarily when performed three hours before bedtime [47].

Nighttime physical exercise, especially high-intensity interval training, could affect sleep quality if performed less than an hour before bedtime [48]. Therefore, the timing of exercise is vital, with some evidence suggesting that morning or afternoon exercise may be more beneficial for sleep than nighttime or evening vigorous exercise [48,49]. However, depending on the degree of endurance the body is used to, the effect might be different. It was found that for endurance-trained runners, high-intensity exercise does not interrupt and may even benefit future nocturnal sleep when conducted in the early evening [50]. Other than timing, the effectiveness of physical activity in improving sleep quality is influenced by various factors, such as type and duration. Studies have suggested that moderate-intensity aerobic exercise, strength training, and mind-body physical exercises like yoga and tai chi may improve sleep quality [51]. Studies conducted to evaluate the effect of yoga, tai chi, and qigong on sleep quality found that they improve sleep outcomes and decrease insomnia [51-53]. Our findings show that these types of exercises had more significant effects on sleep outcomes than other types. We also found that duration plays a role in the effectiveness of physical activities in sleep improvement, together with timing and intensity. High intensity exercises for more than 90 minutes in the evening has been associated with difficulty in sleeping, while light exercises of 10 minutes per day in the morning improved sleep quality [48,49].

The key to obtaining the advantages of physical exercise is consistency and regularity. Individuals who engage in regular physical activities and follow a consistent regimen might see considerable benefits in their overall health and well-being [15-18]. There is better sleep efficiency and higher overall sleep satisfaction among people who perform regular physical activities [54]. The WHO recommends 150-300 minutes of moderate-intensity aerobic physical exercises per week for adults [44], equivalent to 30-minute exercises three to six times a week. Our review’s findings showed that aerobic exercise four to seven times a week and moderate intensity exercise three times a week were associated with better sleep quality.

Studies have found that aerobic exercises improve sleep quality and are effective in patients with insomnia [21,51]. However, unlike healthy people, exercising six or more times a week could lead to poor sleep quality among insomnia patients. Three months or less of physical exercise was more effective in improving sleep quality than more than three months of physical exercise. This indicates that using physical activities in managing patients with insomnia should be tailored to the particularities of these patients. More research is needed to explore how physical activities impact these patients. Physical exercise can act as a natural sleep aid, promoting relaxation, and reducing the hyperarousal associated with insomnia supporting the integration of physical exercise in the treatment modalities of insomnia [55,56]. Physical activities help improve sleep quality more effectively in children and older people than young adults and adolescents, leading to improved cognitive function and physical and psycho-social well-being [44,57,58].

This systematic review has several limitations to consider. There was a lot of diversity among the studies regarding physical activity/exercise definition, study designs, and populations, and there were no consistent criteria for evaluating variables among studies, which might affect identified associations. Articles included in this review did not sufficiently explore the impact of physical activities on other sleep disorders, such as apnea and restless leg syndrome though some looked at insomnia. The systematic review design is prone to selection bias and statistical heterogeneity; therefore, further research is recommended to address these limitations.

## Conclusions

Regular physical activity can lead to improved sleep quality, reduced sleep latency, and better overall sleep quality. Moreover, physical activity has shown promise in managing sleep disorders like insomnia. Regular moderate-intensity physical activities are the most effective, while high-intensity physical activities, especially in the evening or close to bedtime, may lead to difficulty sleeping. Other factors influencing the effectiveness of physical activities in improving sleep quality include gender, age, activity type, timing, duration, and consistency. Further research is needed to determine the optimal exercise regimens and mechanisms underlying the sleep benefits of physical activity. Nevertheless, promoting regular physical activity can be an effective approach to improving sleep health and overall well-being.
